# High-Fat Feeding in Time-Dependent Manner Affects Metabolic Routes Leading to Nervonic Acid Synthesis in NAFLD

**DOI:** 10.3390/ijms20153829

**Published:** 2019-08-05

**Authors:** Karolina Konstantynowicz-Nowicka, Klaudia Berk, Adrian Chabowski, Irena Kasacka, Patrycja Bielawiec, Bartłomiej Łukaszuk, Ewa Harasim-Symbor

**Affiliations:** 1 Department of Physiology, Medical University of Bialystok, 15-089 Bialystok, Poland; 2 Department of Histology and Cytophysiology, Medical University of Bialystok, 15-089 Bialystok, Poland

**Keywords:** diacylglycerol, fatty acids, diet and dietary lipids, liver, fatty acid metabolism, nervonic acid, elongation, desaturation, lipogenesis

## Abstract

Nonalcoholic fatty liver disease (NAFLD) is characterized by excessive lipid accumulation in the liver. The disturbances in the fatty acid composition of stored lipids are more important than the lipid species itself, which may influence the overall effect caused by these molecules. Thus, uncovering time-dependent changes in the fatty acid composition of accumulated lipid fractions after a high fat diet seems to be a new marker of NAFLD occurrence. The experiments were conducted on high fat fed Wistar rats. The blood and liver samples were collected at the end of each experimental week and used to assess the content of lipid fractions and their fatty acid composition by gas liquid chromatography. The expression of proteins from lipid metabolism pathways and of fatty acid exporting proteins were detected by Western blotting. In the same high fat feeding period, decreased de novo lipogenesis, increased β-oxidation and lipid efflux were demonstrated. The observed effects may be the first liver protective mechanisms against lipotoxicity. Nevertheless, such effects were still not sufficient to prevent the liver from proinflammatory lipid accumulation. Moreover, the changes in liver metabolic pathways caused the plasma nervonic acid concentration in sphingomyelin to decrease simultaneously with NAFLD development, which may be a steatosis occurrence prognostic marker.

## 1. Introduction

Nonalcoholic fatty liver disease (NAFLD) is an important health problem in Western society characterized by the excessive content of triacylglycerols in the cytoplasm of hepatocytes exceeding 5% of their volume [1,2]. NAFLD occurrence is strongly associated with obesity and type 2 diabetes [3]. Some obese individuals exhibit increased circulating levels of free fatty acids (FFAs) in plasma whose sources are either: unhealthy diet, augmented adipose tissue lipolysis, or de novo lipogenesis [4,5]. The elevated levels of FFAs reaching the liver override its oxidation capacity and are esterified mainly to triacylglycerol (TAG) and diacylglycerol (DAG) lipid fractions, which are accumulated in hepatocytes [6]. It is still unclear whether TAG deposition is the liver’s protective mechanism against lipotoxicity or is a culprit in NAFLD development [7]. Studies conducted by Listenberger et al. revealed that increased TAG accumulation during lipid oversupply state is a cellular defence against lipotoxic effects [8]. Although this lipid fraction may also be a source for other more biologically active lipids, such as DAG, which may cause insulin resistance development and NAFLD deterioration [5]. In our opinion more important is the fatty acid composition rather than the lipid species itself, which may influence the overall effect caused by lipids. It may explain why some obese patients do not develop insulin resistance and NAFLD [9]. Interestingly, fatty acids composition present in diet reflects TAG and DAG composition accumulated in the liver [10]. The Western style diet, which is rich in saturated fatty acids (SFA), leads to the deposition of mainly palmitic acid in different lipid fractions. However, the amount of other fatty acids is also important as it may indicate the direction of metabolic changes. The nervonic acid (NA; C 24:1), being a monounsaturated derivative of lignoceric acid, has been claimed to have beneficial effects on risk factors connected with obesity and diabetes [11,12]. As reported earlier, the content of this fatty acid was affected in peroxisomal disorders, diabetes, and undernourishment [13,14,15]. However, no information is available regarding the alteration in nervonic acid content during NAFLD development. The nervonic acid is considered to be a predicting factor for NAFLD occurrence and progression. The present study concerns the relationship between time dependent high fat feeding, NA content in various lipid fractions and NAFLD development. Such measurements are necessary to elucidate changes in liver metabolic routes and lipid fraction content during overnutrition, which may help to find new potential biomarkers associated with obesity and NAFLD.

## 2. Results

### 2.1. Changes in Liver Lipid Concentration

**Figure 1:**
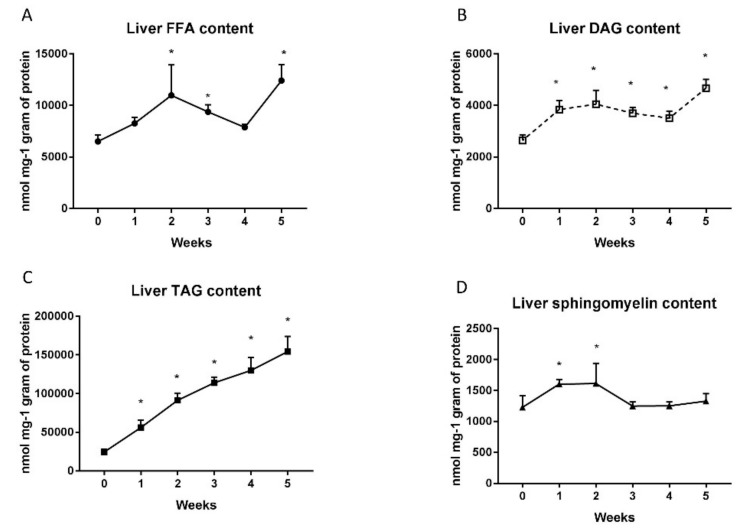
Liver contents of free fatty acids (**A**), diacylglycerols (**B**), triacylglycerols (**C**) and sphingomyelin (**D**) in control group (0 week) and high fat diet (HFD) group at the end of each experimental week (1, 2, 3, 4 and 5). The liver homogenates were prepared as described in detail in the materials and methods section. Total lipid content was measured by the gas-liquid chromatography (GLC) method. The data are expressed as the mean ± S.D. and are based on six independent determinations (*n* = 6). * *p* < 0.05 significant difference vs control group (0 week).

The level of FFA in liver homogenates after high fat diet (HFD) administration rise significantly in the second (2nd week: + 68%; *p* < 0.001; Figure 1A), third (3rd week: + 44%; *p* < 0.05; Figure 1A) and fifth (5th week: + 90%; *p* < 0.0001; Figure 1A) week of feeding in comparison to the standard chow control group. Moreover, a high fat diet increased considerably the DAG (1st week: +45.5%; *p* < 0.005; 2nd week: +53%; *p* < 0.02; 3rd week: +40%; *p* < 0.001; 4th week: +32%; *p* < 0.001; 5th week: +76%; *p* < 0.001; Figure 1B) and TAG (1st week: +129.5%; *p* < 0.005; 2nd week: +273.7%; *p* < 0.001; 3rd week: +366.2%; *p* < 0.001; 4th week: +431.9%; *p* < 0.001; 5th week: +531.6%; *p* < 0.001; Figure 1C) contents in each week of animal feeding. Interestingly, the level of sphingomyelin in the liver of HFD group was elevated only in the first (1st week: + 30.6%; *p* < 0.05; Figure 1D) and the second (2nd week: + 31.5%; *p* < 0.05; Figure 1D) weeks of the diet.

### 2.2. Changes in the Plasma Lipid Concentrations 

**Figure 2:**
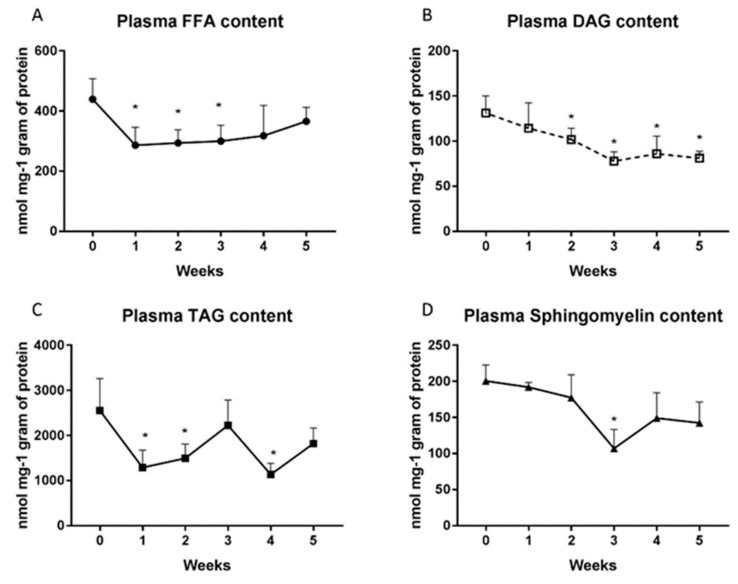
Plasma contents of free fatty acids (**A**), diacylglycerols (**B**), triacylglycerols (**C**) and sphingomyelin (**D**) in control group (0 week) and HFD group at the end of each experimental week (1, 2, 3, 4 and 5). The plasma was prepared as described in detail in the ‘Materials and methods’ section. Total lipid content was measured by GLC method. The data are expressed as the mean ± S.D. and are based on six independent determinations (*n* = 6). * *p* < 0.05 significant difference vs control group (0 week).

The HFD feeding reduced markedly the level of FFA in the first, second and third (1st week: −64.9%; 2nd week: −33.2%; 3rd week: −31.2%; *p* < 0.05; Figure 2A) weeks compared to the control group. The DAG content declined in HFD groups throughout the five-week period, starting from the second week (2nd week: −22.5%; 3rd week: −40.7%; 4th week: −34.3%; 5th week: −38%; *p* < 0.05; Figure 2B). Furthermore, TAG concentrations decreased significantly in the first, second and fourth weeks in the HFD rats (1st week: 50%; 2nd week: −41.5%; 4th week: −55.6%; *p* < 0.05; Figure 2C). Diet-induced elevation of plasma sphingomyelin content was considerable only in the third week (3rd week: −46.7%; *p* < 0.001; Figure 2D).

### 2.3. Changes in the Expression of Fatty Acid Transporters

**Figure 3:**
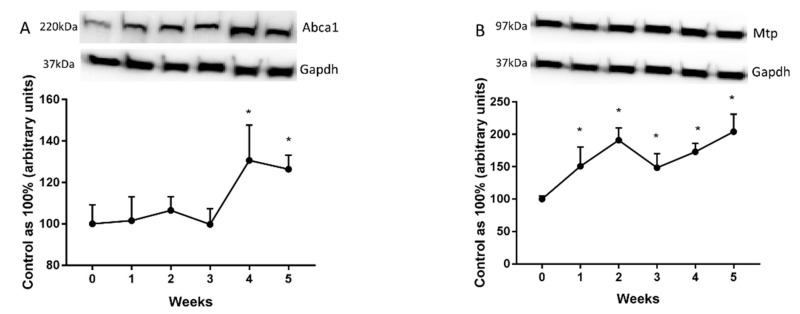
Representative Western blots showing the expression of proteins involved in fatty acids transport outside the cell, i.e., ATP-binding cassette transporter A1 (Abca1) (**A**) and microsomal triacylglycerol transfer protein (Mtp) (**B**) in the liver homogenates from control group (0 week) and HFD group at the end of each experimental week (1, 2, 3, 4, and 5). The protein expression was measured using Western blot method as it was described in ‘Materials and methods’ section. The data are expressed as the mean ± S.D. and are based on six independent determinations (*n* = 6). * *p* < 0.05 significant difference vs control group (0 week). The samples derived from the same experiment and blots were processed in parallel.

We showed that ATP-binding cassette transporter A1 (Abca1) expression was elevated significantly only at the end of our study (4th week: +40.6%; 5th week: +26.4%; *p* < 0.05; Figure 3A). However, we revealed a substantial rise in microsomal triacylglycerol transfer protein (Mtp) expression provoked by HFD in all the examined groups (1st week: +50.7%; 2nd week: +91%; 3rd week: +48.4%; 4th week: +72.9%; 5: +104%; *p* < 0.05; Figure 3B) in comparison with the control group.

### 2.4. Changes in the Expression of Proteins Directly Involved in Lipid Metabolism 

**Figure 4:**
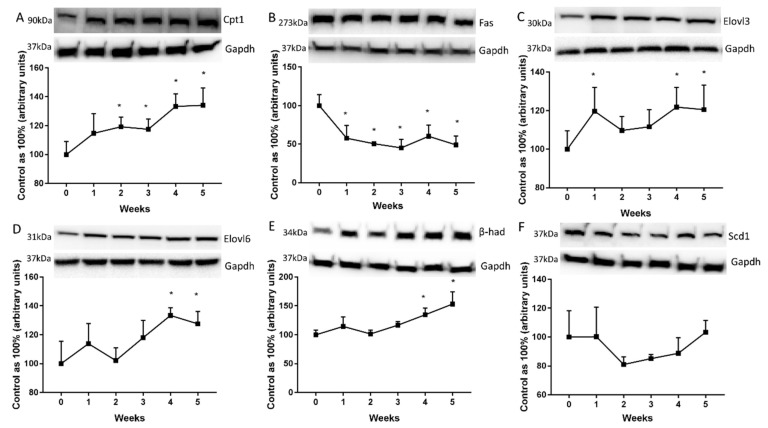
Representative Western blots showing the expression of proteins involved in fatty acids metabolism, i.e. carnitine palmitoyltransferase 1 (Cpt1) (**A**), fatty acid synthase (Fas) (**B**), elongase 3 (Elovl3) (**C**), elongase 6 (Elovl6) (**D**), beta-hydroxyacyl-CoA dehydrogenase (β-had) (**E**) and stearoyl-CoA desaturase 1 (Scd1) (**F**) in the liver homogenates from control group (0 week) and HFD group at the end of each experimental week (1, 2, 3, 4 and 5). The protein expression was measured using Western blot method as it was described in ‘Materials and methods’ section. The data are expressed as the mean ± S.D. and are based on six independent determinations (*n* = 6). * *p* < 0.05 significant difference vs control group (0 week). The samples derived from the same experiment and blots were processed in parallel.

The expression of carnitine palmitoyltransferase 1 (Cpt1) was markedly increased from the second week to the end of HFD feeding (2nd week: +19.2%; *p* < 0.05, 3rd week: +17.5%; *p* < 0.05, 4th week: +33.3%; *p* < 0.001; 5th week: +34.1%; *p* < 0.001; Figure 4A). To our surprise, the fatty acid synthase (Fas) expression in liver homogenates decreased markedly in each week, except for the first week of high fat diet. (2nd week: −42.3%; 3rd week: −49.4%; 4th week: −54.9%; 5th week: −51%; *p* < 0.001; Figure 4B). However, the high fat feeding provoked a rise of elongase 3 (Elovl3) and elongase 6 (Elovl6) expression as compared with the standard chow fed group (Elovl3: 2nd week: +19.7%; *p* < 0.05; 4th week: +21.8%; *p* < 0.05; 5th week: +20.5%; *p* < 0.05; Figure 4C Elovl6: 4th week: +33.4%; *p* < 0.05; 5th week: +27.6%; *p* < 0.02; Figure 4D). Furthermore, we observed a significant elevation of beta-hydroxyacyl-CoA dehydrogenase (β-had) expression at the end of our study in comparison with the control group (4th week: +34.5%; *p* < 0.05; 5th week: +53.1%; *p* < 0.001; Figure 4E). Decreased expression of stearoyl-CoA desaturase 1 (Scd1) was observed in the liver of HFD rats but it did not reach a significant level (Figure 4F).

### 2.5. Changes in the Nervonic Acid Concentration in the Liver and Plasma 

**Figure 5:**
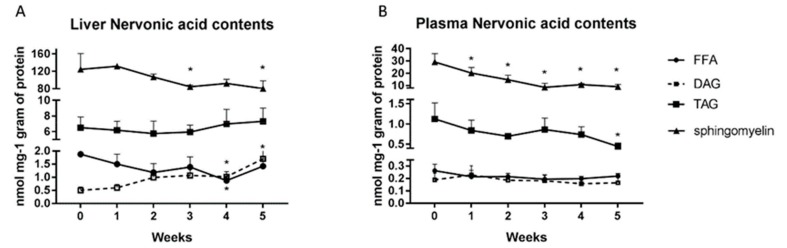
Content of nervonic acid in free fatty acids, diacylglycerols, triacylglycerols and sphingomyelin fraction in the liver homogenates (**A**) and plasma (**B**) from control group (0 week) and HFD group at the end of each experimental week (1, 2, 3, 4 and 5). The nervonic acid content in particular fatty acid fraction was measured by GLC method. The data are expressed as the mean ± S.D. and are based on six independent determinations (*n* = 6). * *p* < 0.05 significant difference vs control group (0 week).

In liver homogenates, we showed a decrease of nervonic acid levels in FFA fraction in the fourth week (4th week: −53.4%; *p* = 0.02; Figure 5A) and in sphingomyelin fraction in the third (3rd week: −32%; *p* < 0.05; Figure 5A) and fifth weeks (5th week: −35.4%; *p* < 0.05; Figure 5A) of HFD feeding. In contrast, the high fat diet elevated significantly the nervonic acid content in DAG fraction but only at the end of our study (4th week: +104.8%; *p* < 0.02, 5th week: +240.9%; *p* < 0.04; Figure 5A).

In plasma, we observed a substantial reduction in the nervonic acid content in sphingomyelin fraction in each week of high fat diet administration (1st week: −33.3%; 2nd week: −51.8%; 3rd week: −74.9%; 4th week: −65.4%; 5th week: −71.4%; *p* < 0.0001; Figure 5B). Furthermore, we revealed a trend towards a reduction in plasma TAG nervonic acid level during HFD feeding, but only in the fifth week was significance reached (5th week: −62.1%; *p* < 0.05; Figure 5B).

### 2.6. Changes in the Elongation and Desaturation Ratio 

**Figure 6:**
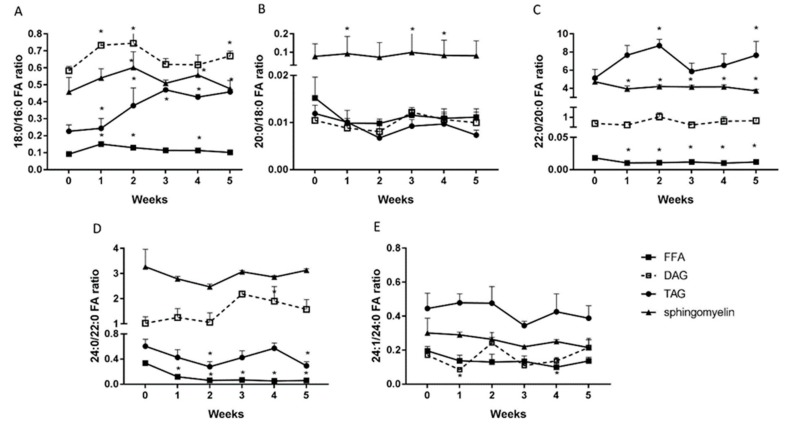
Elongation ratio of the stearic acid/ palmitate (18:0/16:0) (**A**), arachidic acid/stearic acid (20:0/18:0) (**B**), behenic acid/arachidic acid (22:0/20:0) (**C**), lignoceric acid/behenic acid (24:0/22:0) (**D**) and the ratio of unsaturated to saturated fatty acids (desaturation index), i.e., nervonic acid/lignoceric acid (24:1/24:0) (**E)** in triacylglycerols, diacylglycerols, free fatty acids, and sphingomyelin in liver homogenates from control group (0 week) and HFD group at the end of each experimental week (1, 2, 3, 4 and 5). Elongation and desaturation ratios were calculated based on fatty acid composition measured by GLC method. The data are expressed as the mean ± S.D. and are based on six independent determinations (*n* = 6). * *p* < 0.05 significant difference vs control group (0 week).

The 18:0/16:0 ratio was elevated during high fat diet feeding in FFA fraction in each week of our study (1st week: 0.24; 2nd week: 0.38; 3rd week: 0.47; 4th week: 0.43; 5th week: 0.46; *p* < 0.001; Figure 6A), and in DAG and TAG fractions in the first, second and fifth weeks compared with the standard chow fed group (DAG: 1st week: 0.73; 2nd week: 0.74; 5th week: 0.7; TAG: 1st week: 0.15; 2nd week: 0.13; 5th week: 0.11; *p* < 0.01; Figure 6A). In the sphingomyelin, we observed a significant increase of the 18:0/16:0 ratio in the second and the fifth weeks in comparison with the control group (2nd week: 0.6; 5th week: 0.48; *p* < 0.05; Figure 6A). The 20:0/18:0 ratio rose only in the sphingomyelin fraction, in the first, third, and fourth weeks, which was statistically significant compared to the standard fed chow (1st week: 0.09; 3rd week: 1; 5th week: 0.09; *p* < 0.05; Figure 6B). What is more, we showed a significant rise in FFA’s 22:0/20:0 ratio during the second, third, and fifth week of the high fat diet (2nd week: 7.6; 3rd week: 8.6; 5th week: 7.6; *p* < 0.05; Figure 6C). In contrast, the TAG’s and sphingomyelin’s 22:0/20:0 ratios decreased in each week of our study in comparison with the control group (TAG: 1st, 2nd, 3rd, 4th, 5th week: 0.01; *p* < 0.05; Sphingomyelin: 1st week: 3.2; 2nd week: 4.1; 3rd week: 4.1; 4th week: 4.1; 5th week: 3.7; *p* < 0.05; Figure 6C).

The DAG 24:0/22:0 ratio was markedly elevated in the fourth week of our study (4th week: 2.2; *p* < 0.01; Figure 6D). In contrast, the 24:0/22:0 ratio in FFA and TAG fractions were reduced in comparison with the standard chow fed group (FFA: 3rd week: 0.3; 5th week: 0.3; TAG: 1st week: 0.11; 2nd week: 0.06; 3rd week: 0.07; 4th week: 0.05; 5th week: 0.06; *p* < 0.001; Figure 6D). The HFD induced the decrease in DAG’s 24:1/24:0 ratio in the first week (1st week: 0.08; *p* < 0.05; Figure 6E) as well as the drop in FFA’s 24:1/24:0 ratio in the fourth week (4th week: 0.1; *p* < 0.01; Figure 6E) of feeding.

### 2.7. Changes in the De Novo Lipogenesis Ratio (16:0/18:2 n-3 Ratio), the Activities of n-3 (20:5+22:6)/18:3) and n-6 Pathways (20:4/18:2 n-6). 

**Figure 7:**
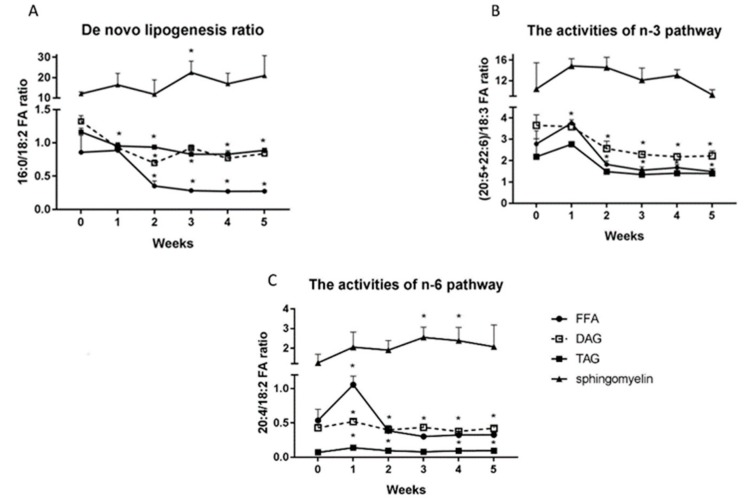
De novo lipogenesis ratio (16:0/18:2) (**A**), the activities of n-3 pathway ((20:5 + 22:6)/18:3) (**B**) and the activities of n-6 pathway (20:4/18:2) (**C**) in triacylglycerols, diacylglycerols, free fatty acids and sphingomyelin fractions in liver homogenates from control group (0 week) and HFD group at the end of each experimental week (1, 2, 3, 4, and 5). Lipogenesis ratio and activities of *n*-3 and *n*-6 pathways were calculated based on fatty acid composition measured by GLC method. The data are expressed as the mean ± S.D. and are based on six independent determinations (*n* = 6). * *p* < 0.05 significant difference vs control group (0 week).

The HFD caused a significant reduction of FFA’s 16:0/18:2n-3 ratio from the second week to the end of our study (2nd week: 0.35; 3rd week: 0.28; 4th week: 0.27 5th week: 0.27; *p* < 0.05; Figure 7A) compared with the control group. Similarly, the DAG and TAG de novo lipogenesis ratio decreased significantly throughout the HFD feeding, and the decline was more pronounced in the fifth week of our study (DAG: 1st week: 0.92; 2nd week: 0.69; 3rd week: 0.92; 4th week: 0.76; 5th week: 0.83; TAG: 1st week: 0.95; 2nd week: 0.93; 3rd week: 0.82; 4th week: 0.83 5th week: 0.88; *p* < 0.05; Figure 7A). In contrast, the 16:0/18:2n-3 ratio in sphingomyelin fraction elevated markedly only in the third week of HFD (3rd week: 22.4; *p* < 0.05; Figure 7A). The FFA’s (20:5+22:6)/18:3 ratio increased considerably in the first week of HFD feeding and then decreased from the second week to the end of our study (1st week: 3.6; 2nd week: 1.8; 3rd week: 1.5; 4th week: 1.6; 5th week: 1.5; *p* < 0.01; Figure 7B). Whereas, the high fat diet provoked the significant reduction of the DAG’s (20:5+22:6)/18:3 ratio from the second week of our study (DAG: 2nd week: 2.5; 3rd week: 2.3; 4th week: 2.1; 5th week: 2.22; *p* < 0.001; Figure 7B).

The high fat diet provoked the increase in FFA’s 20:4/18:2 n-6 ratio in the first week and the decrease in this ratio from the third week of our study, which was statistically significant compared to the control group (FFA: 1st week: 1.05; 3rd week: 0.3; 4th week: 0.32; 5th week: 0.32; *p* < 0.05; Figure 7C). The DAG 20:4/18:2 n-6 ratio increased considerably only in the first week of feeding (1st week: 0.5; *p* < 0.05; Figure 7C). Furthermore, we observed a significant increase in TAG 20:4/18:2 n-6 ratio in all HFD rats, with the exception of the third week in comparison with the standard chow group (1st week: 0.14; 2nd week: 0.1; 4th week: 0.1; 5th week: 0.1; *p* < 0.05; Figure 7C). In the case of sphingomyelin fraction, we revealed a significant increase of 20:4/18:2 n-6 ratio only in the third and fourth weeks of high fat feeding (3rd week: 2.5; 4th week: 2.4; *p* < 0.05; Figure 7C).

### 2.8. Hepatic Histological Changes

**Figure 8:**
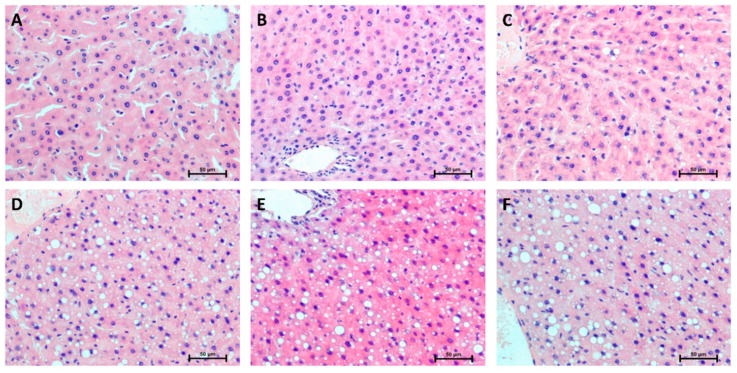
Representative histological images showing hematoxylin–eosin (H + E) staining of the liver from control group (0 week, **A**) and HFD group at the end of the first (**B**), second (**C**), third (**D**), fourth (**E**) and fifth (**F**) experimental week. The data are based on six independent determinations (*n* = 6) of the same fragment of the lobe from each rat. Original magnification of 200× and the length of the calibration bar is 50 µm.

**Figure 9:**
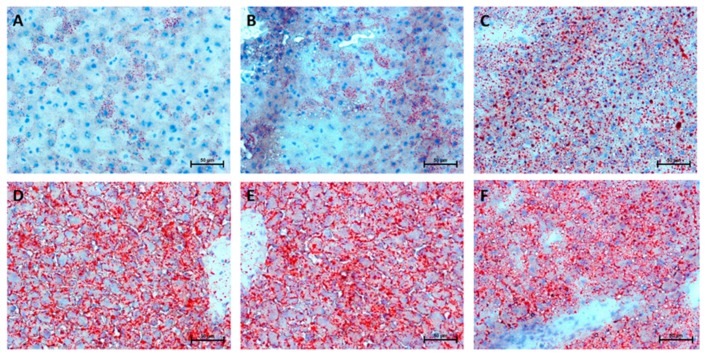
Representative histological images of the liver sections showing lipid droplets accumulation stained by Oil Red O from control group (0 week, **A**) and HFD group at the end of the first (**B**), second (**C**), third (**D**), fourth (**E**) and fifth (**F**) experimental week. The data are based on six independent determinations (*n* = 6) of the same fragment of the lobe from each rat. Original magnification of 200× and the length of the calibration bar is 50 µm.

**Figure 10:**
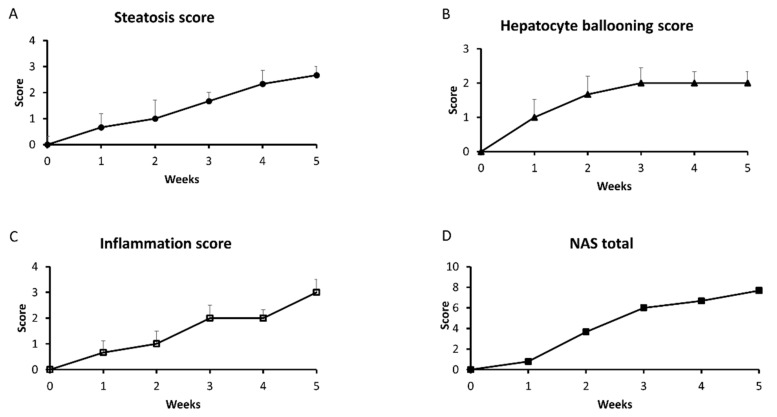
NAFLD activity score of the liver sections, i.e. liver steatosis score (**A**), hepatocyte ballooning score (**B**), inflammation score (**C**) and NAS total (**D**) from control group (0 week) and HFD group at the end of each experimental week (1, 2, 3, 4 and 5). Histological images were used for the scoring system as described in the ‘Materials and methods’ section.

Representative histological images of selected liver sections stained with hematoxylin and eosin are shown in Figure 8. Oil Red O staining of liver sections showed that excessive lipid accumulation (more than 5% of cell size) increased linearly during five weeks of HFD (Figure 9), but was not seen in the 0 week considered as a control group (steatosis score: 0 week: 0,33; 1st week: 0,67; 2nd week: 1,00; 3rd week: 1,67; 4th week: 2,33; 5th week: 2,67; Figure 10A and Figure 9A–F). At the first week of HFD feeding, an increased steatosis score was accompanied by the rise of ballooning cell numbers with the highest number in the last week (1st week: 1,00; 2nd week: 1,67; 3rd week: 2,00; 4th week: 2,00; 5th week: 2,00; Figure 10B and Figure 8A–F). Hepatic inflammation occurred in the third week of feeding with a very mild increase during the first and second weeks (1st week: 0.67; 2nd week: 1.00; 3rd week: 2.00; Figure 10C and Figure 8A–F). At the end of fifth week, HFD induced prominent inflammatory cell infiltration and moderate hepatocyte necrosis (5th week: 3.00; Figure 10C and Figure 8F). Using histological images and the NAFLD activity score (NAS) based on steatosis, inflammation and hepatocellular ballooning, we observed that at the third week of HFD, NAFLD occurred with progression to more severe hepatic damage at the end of the study (NAS score: 3rd week: 6.00; 4th week: 6.67; 5th week: 7.67; Figure 10D).

## 3. Discussion

In Western societies shifting away from the healthy lifestyle towards a lack of physical activity, chronic stress and excessive energy intake (particularly dietary fat in highly processed food products) are considered the main factors behind a predisposition to metabolic disorders. A prolonged, increased dietary fat consumption may change lipid composition and fatty acid saturation status, especially in the liver, as it plays a principal role in the lipid metabolism. In our studies we demonstrated time-dependent changes in lipid content and fatty acid composition, which affects NAFLD development. Quite surprising, histologically, were visible changes in lipid droplet amounts and sizes that were observed after one week of a high fat diet. It is possible that so rapidly occurring lipid accumulation is at first a protective mechanism of the liver against lipotoxicity. The observed increase is consistent with increasing FFA, DAG, and TAG content during five weeks of high fat feeding, but only TAG elevation was linear. These findings suggest that NAFLD development was accompanied by the formation of vesiculae and necrotic changes in the last weeks, which appeared during an increased availability of fatty acids in the diet. This was confirmed by NAS score which was above four after three weeks of high fat feeding. In line with these results are studies conducted on rats with diet-induced NAFLD as well as human liver biopsies from patients diagnosed with this disease, whereas in hepatocytes an increased lipid accumulation was also observed [16,17]. Although extracellular fatty acids concentration was previously described, it remained unclear how its composition and amount are changing during NAFLD development. Some findings implied that a greater increase in TAG rather than FFA concentrations in plasma after eight weeks high fat fed rats were caused by the effective conversion of FFA fraction to TAG, which was stored in different tissues such as the liver and adipose tissue. However, the observed changes were time-dependent and after a longer diet exposition an increase in plasma concentration of these fractions changed significantly because of the disability of the liver to synthetize TAG. Studies conducted by Ipsen et al. also revealed that decreased lipid synthesis caused a reduction of TAG and FFA concentration in plasma after a high fat diet [18]. This is in accordance with our studies, which also showed a decreased plasma concentration of all the analysed lipid fractions. This is not surprising, as the decrease of fatty acid synthase expression, as well as de novo lipogenesis ratio in all the fractions were observed. However, we found an increased expression of Abca1 and Mtp, both responsible for lipid efflux to the circulation. We suspect that lipids transported to the blood stream are effectively stored as TAGs in the adipose tissue, which may explain why some obese individuals do not suffer from hyperlipidemia [19]. Surprisingly, we observed a significantly increased expression of Cpt1 and β-had especially at the end of HFD feeding. Probably intensified β-oxidation is one of the cell’s protective mechanisms against excessive lipid accumulation and lipotoxic effects that may be their result. By contrast, the studies conducted by Liu et al. revealed a reduced expression of Cpt in the HFD group [20]. One possible explanation for this discrepancy is that we were focused on the short term effects of a five-week HFD contrary to the 24 weeks of high fat feeding conducted by those researchers [20]. Thus, we may suggest that the liver’s protective mechanisms, which occur at the beginning of excessive lipid availability are: diminished lipid synthesis, increased β-oxidation, and intensified lipid efflux. All those processes create the basis for lipid redistribution among already existing lipid fractions. In our opinion, it is extremely important to take a closer look into lipid composition to predict what type of negative changes may occur. Thus, in our research we investigated time-dependent changes in nervonic acid concentration in different lipid fractions. In the studies conducted by Yamazaki et al. a decrease in total serum nervonic acid concentration in metabolic syndrome patients was observed [21]. Consistent with this data are studies in which an inverse relationship between nervonic acid content in serum with waist circumference and TNF-α concentration indicated the progression of obesity-related diseases and metabolic dysregulation [22]. In line with those results, we found a decreased concentration of the plasma nervonic acid in sphingomyelin fraction starting with the first week of high fat feeding with the most pronounced changes in the last two weeks of the diet. Moreover, NA concentration in the liver sphingomyelin was markedly lower only in the third and fifth weeks of HFD. However, the liver DAG level was substantially increased at the end of feeding. Observed changes were simultaneous with NAFLD development characterized by a NAS score above four at the end of our study. These findings are in accordance with various studies that associated the changes in nervonic acid concentration to diseases like diabetes and obesity, both related to NAFLD development [12]. However, little is known about the factors and metabolic pathways which lead to nervonic acid synthesis especially in the fatty liver. The palmitic acid, the main component of HFD, may be elongated to 18- and more carbon fatty acids by the activity of Elovl6 and after this—Elovl3. Next, the products of elongation can be desaturated by stearylo-CoA desaturase 1, which is the last step in the nervonic acid synthesis pathway [23]. The data presented herein show the increased expression of elongases in the fourth and fifth weeks of high fat feeding with an augmentation of the elongation ratio in FFA, DAG, and sphingomyelin fraction in selected fatty acids at the beginning of the metabolic pathway leading to nervonic acid synthesis. Moreover, we observed a trend towards a decrease in desaturation ratio in all lipid fractions and a simultaneous slight decrease in the expression of Scd1. Based on these results, an increased expression of proteins responsible for lipid extension at the end of HFD feeding, not reflected by changes in the elongation products, may be explained by an insufficient feeding period. The changes in protein expression may precede changes in lipid products; thus, a longer exposition time is needed to observe significant manifestation of the elongation process. Supporting this conclusion are studies where mice were administered a high fat diet during the 56-day period. Those animals demonstrated increased elongase activities, which proved that a longer feeding period may be vital for more pronounced changes [24]. As our study results do not show augmentation of the nervonic acid synthesis pathway, we extended our study to explore the fatty acid omega-3 and -6 synthesis route. Our results confirmed that lipids metabolism was redirected to the accumulation of proinflammatory n-6 fatty acids among various lipid fractions. It was seen in the increase of 20:4/18:2 ratio especially in sphingomyelin and TAG fractions with a simultaneous decrease of (20:5+22:6)/18:3 ratio in FFA and DAG as the indicator of n-3 pathway activity. It is in accordance with the studies conducted by Wang et al., who found that an increased level of n-6 fatty acid is a predisposing factor for chronic inflammation [25].

In conclusion, we may suspect that a high fat diet gradually affected hepatic lipid metabolism by shifting away from the synthesis of beneficial fatty acids, like nervonic acid, towards the excessive accumulation of proinflammatory lipids, especially in TAG fraction. However, our study clearly showed the simultaneous progression of protective mechanisms against lipotoxicity expressed as excessive accumulation of TAG in the liver, intensified lipid β-oxidation and efflux to the circulation. Moreover, the observed redistribution in fatty acids among lipid fractions and decrease in nervonic acid concentration, especially in plasma sphingomyelin, reflecting the development and progression of NAFLD, has a valuable potential to become a new prognostic marker for the occurrence of this disease.

## 4. Materials and Methods 

### 4.1. Animals and Study Design

The following experimental procedures were conducted on male Wistar rats (six rats in the each group, 100–150 g of initial body weight) kept in approved animal holding facilities (at 22 ± 2 °C, on a 12 h/12 h light–dark cycle, with unrestricted access to water and to commercial chow). The rats were randomly divided into two groups: (1) control receiving a standard chow diet, (2) HFD group fed ad libitum on a rodent diet rich in fatty acids (kcal distribution = 60% fat, 20% carbohydrate and 20% protein; Research Diet Inc., New Brunswick, NJ, USA, cat no. D12492). In our experiment we set two control groups receiving a standard diet: one at the 1st week and the second at the 5th week of the study. Because no significant changes between these control groups were observed in the same measurements, the results were referred to as one control group (0 week). The animals from control groups, as well as HFD fed groups, at the end of each experimental week (1, 2, 3, 4 and 5) (fasted overnight) were anaesthetized by intraperitoneal injection of pentobarbital in the dose of 80 mg/kg of body weight. Samples of the liver were excised, immediately frozen—clamped with aluminum tongs precooled in liquid nitrogen and stored in −80 °C until further analyses. Blood samples were collected through the inferior vena cava to heparinized tubes, centrifuged, and the plasma was separated. All experimental procedures and the number of animals were approved by the Ethics Committee on Animal Care at the Medical University of Bialystok (28 May 2008, approval number: 32/2008). Moreover, all methods used in our experiments were performed in accordance with the relevant guidelines and regulations.

### 4.2. Serum and Liver Lipid Analysis 

Plasma and liver concentrations of the individual fatty acid methyl esters were extracted with a chloroform-methanol solution using the Folch [26] method and separated into free fatty acids (FFA), DAG, TAG, and sphingomyelin fractions by thin-layer chromatography (TLC) [27]. Subsequently, individual fatty acid fractions were methylated in 14% methanol solution in boron trifluoride and quantified according to the retention times of standards with the use of the gas-liquid chromatography procedure (GLC Hewlett-Packard, Palo Alto, CA, USA, 5890 Series II gas chromatography HP- innowax capillary column equipped with a flame ionization detector) as described previously in details [27]. Furthermore, based on fatty acid composition, we calculated the elongation ratio (18:0/16:0; 20:0/18:0; 22:0/20:0; 24:0/22:0), desaturation ratio (24:1/24:0), de novo lipogenesis ratio (16:0/18:2n-3), the activities of the n-3 (20:5 + 22:6)/18:3) and n-6 pathway (20:4/18:2 n-6) in the particular lipid fraction. The amount of FFA, DAG, TAG and sphingomyelin contents were estimated as the sum of the individual fatty acid species of the assessed fractions, and they were expressed in nanomoles per gram of tissue.

### 4.3. Immunoblotting

The expression of proteins directly involved in lipogenesis (Fas; Cell Signaling, Beverly, MA, USA), oxidation pathway (Cpt1, β-had; Santa Cruz Biotechnology, Dallas, TX, USA) and the process of desaturation and elongation (Elovl3, Elovl6, Scd1; Santa Cruz Biotechnology) as well as fatty acid exporting proteins: Abca1 (Thermo Scientific, Waltham, MA, USA) and Mtp (Santa Cruz Biotechnology) were detected by routine Western Blotting as previously described in details by Konstantynowicz-Nowicka et al. [28]. Briefly, protein concentration was determined using bicinchonic acid method (BCA) with bovine serum albumin (BSA) as a standard. Signals obtained by immunoblotting were quantified densitometrically using a ChemiDoc visualization system (Bio Rad, Warsaw, Poland). Equal protein loading was confirmed using Ponceau S staining. The expression of all the proteins was standardized to the Gapdh (Santa Cruz Biotechnology) expression and the control was set at 100%.

### 4.4. Liver Histopathology

Samples of the liver (the same fragment of the lobe from each rat) were collected for histologic studies. They were fixed in 10% buffered formalin, and processed routinely for embedding in paraffin. Sections were cut at 4 µm in thickness and stained with hematoxylin–eosin (H + E). Lipid droplets in liver tissues were determined by Oil Red O staining. Freshly picked fragments of liver were placed in the Tissue-Tek® O.C.T.™ (Sakura Finetek, Alphen aan den Rijn, The Netherlands), then frozen at ™30 °C. The 7-μm thick sections were cut on the frozen microtome and placed on adhesive slides. The sections were incubated in propylene glycol for 2 min. and, subsequently, in Oil Red O solution for 6 min. Next, the tissue sections were differentiated in 85% propylene glycol for 1 min., rinsed twice in water and incubated in hematoxylin for 1–2 min. The sections were then rinsed in tap water by customary procedure and coversliped with an aqueous mounting medium. The results of staining were submitted for evaluation in an Olympus BX41 microscope with an Olympus DP12 camera (Hamburg, Germany) under a magnification of 200 × (20 × lens and 10 × eyepiece).

Hepatic steatosis, inflammation, and hepatocellular ballooning were assessed by four independent pathologists unaware of the type of experimental group. They scored NAFLD diagnosis using NAS (NAFLD activity score ranged totally from 0 to 8) [29] as the most popular grading and staging system consisting of three parts as follows:

a)  Steatosis 

0 for < 5% of hepatocytes steatotic;

1 for 5–33% of hepatocytes steatotic;

2 for 34–66% of hepatocytes steatotic;

3 for >66% of hepatocytes steatotic.

b)  Hepatocyte ballooning

0 for none balloon hepatocytes;

1 for few balloon hepatocytes;

2 for many balloon hepatocytes.

c)  Inflammation

0 for none inflammatory foci

1 for 1–2 inflammatory foci per ×20 field;

2 for 2–4 inflammatory foci per ×20 field;

3 for >4 inflammatory foci per ×20 field.

A score of >4 with steatosis and hepatocyte ballooning was considered as NAFLD.

### 4.5. Data Analyses 

The data are expressed as the mean and standard deviation. The assumptions of the methods used in our analysis, that is normality of the data distribution (Shapiro-Wilk test) and homogeneity of the variance (Bartlett’s test), were checked. Statistical differences were determined based on the results of one-way ANOVA followed by an appropriate post-hoc test (i.e. pairwise Student’s *t*-test) using GraphPad Prism 7 (San Diego, CA, USA). *p* < 0.05 was accepted as statistically significant in all cases.

## References

[1] Onyekwere C.A., Ogbera A.O., Samaila A.A., Balogun B.O., Abdulkareem F.B. (2015). Nonalcoholic fatty liver disease: Synopsis of current developments. Niger. J. Clin. Pract..

[2] Dassanayake A.S., Kasturiratne A., Rajindrajith S., Kalubowila U., Chakrawarthi S., De Silva A.P., Makaya M., Mizoue T., Kato N., Wickremasinghe A.R. (2009). Prevalence and risk factors for non-alcoholic fatty liver disease among adults in an urban Sri Lankan population. J. Gastroenterol. Hepatol..

[3] Fabbrini E., Magkos F. (2015). Hepatic Steatosis as a Marker of Metabolic Dysfunction. Nutrients.

[4] Cusi K. (2012). Role of obesity and lipotoxicity in the development of nonalcoholic steatohepatitis: Pathophysiology and clinical implications. Gastroenterology.

[5] Birkenfeld A.L., Shulman G.I. (2014). Nonalcoholic fatty liver disease, hepatic insulin resistance, and type 2 diabetes. Hepatology.

[6] Di Minno M.N., Russolillo A., Lupoli R., Ambrosino P., Di Minno A., Tarantino G. (2012). Omega-3 fatty acids for the treatment of non-alcoholic fatty liver disease. World. J. Gastroenterol..

[7] Leamy A.K., Egnatchik R.A., Young J.D. (2013). Molecular mechanisms and the role of saturated fatty acids in the progression of non-alcoholic fatty liver disease. Prog. Lipid Res..

[8] Listenberger L.L., Han X., Lewis S.E., Cases S., Farese R.V., Ory D.S., Schaffer J.E. (2003). Triglyceride accumulation protects against fatty acid-induced lipotoxicity. Proc. Natl. Acad. Sci. USA.

[9] Browning J.D., Szczepaniak L.S., Dobbins R., Nuremberg P., Horton J.D., Cohen J.C., Grundy S.M., Hobbs H.H. (2004). Prevalence of hepatic steatosis in an urban population in the United States: Impact of ethnicity. Hepatology.

[10] Neuschwander-Tetri B.A., Ford D.A., Acharya S., Gilkey G., Basaranoglu M., Tetri L.H., Brunt E.M. (2012). Dietary trans-fatty acid induced NASH is normalized following loss of trans-fatty acids from hepatic lipid pools. Lipids.

[11] Malaisse W.J., Portois L., Sener A., Carpentier Y.A. (2008). Perturbation of 11-eicosenoate metabolism in female diabetic rats. Int. J. Mol. Med..

[12] Oda E., Hatada K., Kimura J., Aizawa Y., Thanikachalam P.V., Watanabe K. (2005). Relationships between serum unsaturated fatty acids and coronary risk factors: Negative relations between nervonic acid and obesity-related risk factors. Int. Heart J..

[13] Sandhir R., Khan M., Chahal A., Singh I. (1998). Localization of nervonic acid beta-oxidation in human and rodent peroxisomes: Impaired oxidation in Zellweger syndrome and X-linked adrenoleukodystrophy. J. Lipid Res..

[14] Seigneur M., Freyburger G., Gin H., Claverie M., Lardeau D., Lacape G., Le Moigne F., Crockett R., Boisseau M.R. (1994). Serum fatty acid profiles in type I and type II diabetes: Metabolic alterations of fatty acids of the main serum lipids. Diabetes Res. Clin. Pract..

[15] Yeh Y.Y. (1988). Long chain fatty acid deficits in brain myelin sphingolipids of undernourished rat pups. Lipids..

[16] Gorden D.L., Myers D.S., Ivanova P.T., Fahy E., Maurya M.R., Gupta S., Min J., Spann N.J., McDonald J.G., Kelly S.L. (2015). Biomarkers of NAFLD progression: A lipidomics approach to an epidemic. J. Lipid Res..

[17] Yuan F., Wang H., Tian Y., Li Q., He L., Li N., Liu Z. (2016). Fish oil alleviated high-fat diet-induced non-alcoholic fatty liver disease via regulating hepatic lipids metabolism and metaflammation: A transcriptomic study. Lipids Health Dis..

[18] Ipsen D.H., Tveden-Nyborg P., Rolin B., Rakipovski G., Beck M., Mortensen L.W., Færk L., Heegaard P.M., Møller P., Lykkesfeldt J. (2016). High-fat but not sucrose intake is essential for induction of dyslipidemia and non-alcoholic steatohepatitis in guinea pigs. Nutr. Metab. (Lond).

[19] Ipsen D.H., Tveden-Nyborg P., Lykkesfeldt J. (2016). Dyslipidemia: Obese or Not Obese-That Is Not the Question. Curr. Obes. Rep..

[20] Liu J., Han L., Zhu L., Yu Y. (2016). Free fatty acids, not triglycerides, are associated with non-alcoholic liver injury progression in high fat diet induced obese rats. Lipids Health Dis..

[21] Yamazaki Y., Kondo K., Maeba R., Nishimukai M., Nezu T., Hara H. (2014). Proportion of nervonic acid in serum lipids is associated with serum plasmalogen levels and metabolic syndrome. J. Oleo. Sci..

[22] Pickens C.A., Sordillo L.M., Comstock S.S., Harris W.S., Hortos K., Kovan B., Fenton J.I. (2015). Plasma phospholipids, non-esterified plasma polyunsaturated fatty acids and oxylipids are associated with BMI. Prostaglandins Leukot Essent Fatty Acids.

[23] Softic S., Cohen D.E., Kahn C.R. (2016). Role of Dietary Fructose and Hepatic De Novo Lipogenesis in Fatty Liver Disease. Dig. Di.s Sci..

[24] Da Silva-Santi L.G., Antunes M.M., Caparroz-Assef S.M., Carbonera F., Masi L.N., Curi R., Visentainer J.V., Bazotte R.B. (2016). Liver Fatty Acid Composition and Inflammation in Mice Fed with High-Carbohydrate Diet or High-Fat Diet. Nutrients.

[25] Wang X.H., Li C.Y., Muhammad I., Zhang X.Y. (2016). Fatty acid composition in serum correlates with that in the liver and non-alcoholic fatty liver disease activity scores in mice fed a high-fat diet. Environ. Toxicol. Pharmacol..

[26] Folch J., Lees M., Sloane Stanley G.H. (1957). A simple method for the isolation and purification of total lipides from animal tissues. J. Biol. Chem..

[27] Chabowski A., Żendzian-Piotrowska M., Konstantynowicz K., Pankiewicz W., Mikłosz A., Łukaszuk B., Górski J. (2013). Fatty acid transporters involved in the palmitate and oleate induced insulin resistance in primary rat hepatocytes. Acta. Physiol. (Oxf).

[28] Konstantynowicz-Nowicka K., Harasim E., Baranowski M., Chabowski A. (2015). New evidence for the role of ceramide in the development of hepatic insulin resistance. PLoS. ONE.

[29] Kleiner D.E., Brunt E.M., Van Natta M., Behling C., Contos M.J., Cummings O.W., Ferrell L.D., Liu Y.C., Torbenson M.S., Unalp-Arida A. (2005). Design and validation of a histological scoring system for nonalcoholic fatty liver disease. Hepatology.

